# Understanding the Values, Qualities, and Preferences of Patients in Their Relationships With Obstetrics and Gynecology Providers: Cross-Sectional Survey With a Mixed Methods Approach

**DOI:** 10.2196/58096

**Published:** 2024-10-16

**Authors:** Ann Blair Kennedy, Anna Tarasidis Harb, Chloe Schockling, Lauren Jackson Ray, Jennifer Palomo, Rebecca Russ-Sellers

**Affiliations:** 1 Department of Biomedical Sciences School of Medicine Greenville University of South Carolina Greenville, SC United States; 2 Family Medicine Department Prisma Health Greenville, SC United States; 3 School of Medicine University of South Carolina Greenville, SC United States; 4 Obstetrics and Gynecology The University of Tennessee Graduate School of Medicine Knoxville, TN United States; 5 Department of Pediatrics University of Pittsburgh School of Medicine Pittsburgh, PA United States; 6 Department of Pathology Massachusetts General Hospital Boston, MA United States

**Keywords:** communication, obstetrics, gynecology, trust, barriers to care, patient-provider relationships

## Abstract

**Background:**

The patient-provider relationship in obstetrics and gynecology (OBGYN) is uniquely complex due to the sensitive nature of examinations and topics. Patients often prefer health care providers who share similar racial, ethnic, gender, or linguistic backgrounds, particularly in sensitive health care situations, to improve communication and comfort, though historically, specific gender preferences for OBGYNs have not been evident.

**Objective:**

This study aims to describe the values, qualities, and preferences of patients in their relationships with OBGYN providers.

**Methods:**

This cross-sectional survey, conducted from October 2019 to December 2019, involved 1039 US OBGYN patients and used a mixed methods approach, integrating quantitative responses and qualitative insights from open-ended questions. Recruitment was facilitated through targeted social media campaigns, and the survey aimed to capture detailed patient preferences and barriers to care by assessing responses on provider traits, patient experiences, and demographic factors. The study’s rigorous data collection and analysis were designed to fill gaps identified in previous research on patient-provider relationships in OBGYN care.

**Results:**

The findings underscore the paramount importance of trust and comfort, with listening skills identified as crucial. A notable finding is the marked preference for same-gender providers, observed in 80.7% (545/675) of participants. Primary barriers to seeking care reported included daily commitments, highlighting the need for accessible and flexible care options.

**Conclusions:**

The study highlights a significant shift from previous scientific findings in patient preferences toward gender concordance and trust in OBGYN settings, diverging from previous research. These results emphasize the need for patient-centered care and tailored communication strategies to enhance patient experiences and outcomes. Future research should focus on diverse populations to broaden the findings’ applicability and explore the impact of recent shifts in health care policies.

## Introduction

The patient-provider relationship in obstetrics and gynecology (OBGYN) presents unique complexities due to the sensitive nature of examinations and discussions. The patient provider relationship is further shaped by increasing emphasis on patient-centered care, which highlights the importance of patient needs, perspectives, beliefs, and values [1,2]. While not always explicitly stated, a closer examination of the existing research reveals potential gaps in the comprehensively evaluating the multifaceted aspects of patient-provider relationships, diverse barriers to care, and evolving patient preferences within the OBGYN context [3-8].

The concept of patient-physician concordance, which emphasizes shared identities such as race, ethnicity, gender, or language, has gained significant attention in health care research. Numerous studies indicate that patients often prefer providers who share similar backgrounds, positing that such shared identities enhance understanding and communication, thereby potentially improving the quality of care [3,5,6,9]. This preference is particularly pronounced in scenarios involving sensitive health matters, where patients may feel more at ease discussing intimate issues with providers who share their cultural background or language [3,6,9-11]. Historically, however, desired traits of OBGYNs expressed by patients did not indicate a gender preference [1-16].

Furthermore, despite strong patient preferences for concordance, conclusive evidence linking patient-provider concordance directly to improved health outcomes remains elusive [5,8,10,17]. This gap highlights a critical need for further research, especially within OBGYN, to elucidate how patient preferences for concordance translate into tangible health outcomes. This inquiry is increasingly relevant given the dynamic shifts in health care delivery, such as the rising number of women in medical professions and the expanding role of nurse practitioners and primary care physicians in providing gynecological care [3,13,18]. This study aims to describe the values, qualities, and preferences of patients in their relationships with OBGYN providers. By documenting these preferences, the research seeks to establish a foundation for future investigations into how these factors might influence patient satisfaction and health outcomes in OBGYN care.

## Methods

### Study Design

This cross-sectional survey, which collected both quantitative and qualitative data, used qualitative insights from open-ended questions for data transformation and validation [19] to investigate factors impacting patient-OBGYN provider relationships in the United States (Multimedia Appendix 1).

### Setting and Participant Recruitment

**Figure 1:**
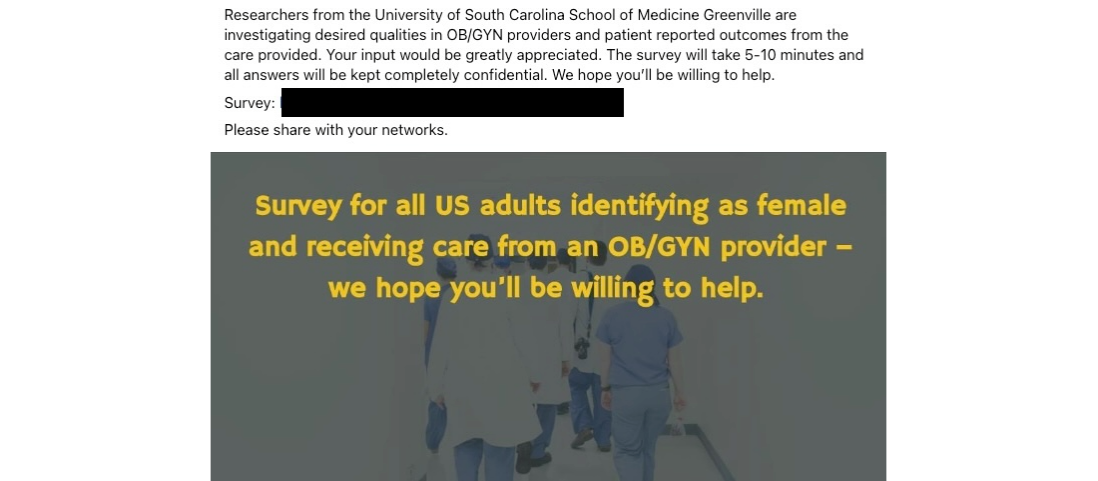
Social media post for recruitment. OB/GYN: Obstetrics and gynecology.

To reduce social desirability bias and elicit truthful responses, an invitation to participate in an anonymous survey was disseminated through social media outlets [20]. Between October 2019 and December 2019, the research team shared posts on the social media platforms such as Facebook, Twitter, Instagram, and LinkedIn through their individual networks and within potential interested groups on Facebook (Figure 1). The recruitment posts asked those who were female and receiving care from an OBGYN provider to complete a confidential 5- to 10-minute survey through a link to a self-administered questionnaire through REDCap (Research Electronic Data Capture; Vanderbilt University) survey software [21]. The posts also asked for others to share the survey within their own networks.

Participants were included in this study if they were aged 18 years or older, consented to participate, had current or previous interactions with an OBGYN provider, and agreed to discuss personal health-related topics. Confidentiality of all responses was ensured to encourage open and honest communication. Though confidentiality was ensured, the survey offered an opportunity for respondents to receive survey analysis results by providing an email address.

### Survey Development

The survey was developed by reviewing existing surveys on OBGYN patient-physician relationships to align the content with current research gaps [1-16]. These key studies highlighted factors influencing the selection of OBGYN providers, such as physician gender, experience, and bedside manner [1-16]. These studies guided the inclusion of questions to assess participants’ preferences and beliefs regarding OBGYN provider characteristics [1-16]. In addition, research on stereotyped beliefs about male and female OBGYNs and patient satisfaction informed the incorporation of items addressing participants’ satisfaction and perceived empathy based on their provider’s gender [16]. In addition, the survey included participant and practice demographics and barriers to care (Multimedia Appendix 1). By integrating these elements, our survey aims to capture a comprehensive understanding of patient preferences and experiences in the context of OBGYN services, addressing gaps identified in previous research.

The specific questions chosen were based on their relevance and proven effectiveness in capturing critical aspects of patient-provider relationships. The studies reviewed provided a robust foundation for identifying key variables and developing a comprehensive survey. By systematically integrating these insights, the final survey instrument was designed to fill identified research gaps and provide valuable data on patient preferences and experiences in OBGYN services.

### Patient and Public Involvement and Engagement

To enhance the survey’s validity and to assist with recruitment procedures, a patient and public engagement group trained in research methodology and communication with researchers assisted the research team. This group of individuals is trusted to critically review research projects and act as coinvestigators throughout the life of the study. As a part of the learning academic health center’s research infrastructure, this group was established in 2016 specifically for the purpose of providing patient and community partner input to co-develop and co-design research. This group included 3 scientists (experienced in health service research, comparative effectiveness research, and social health research), 4 physician representatives, a representative from the patient experience team, and 8-12 patient partners (experts). The patient experts come from diverse backgrounds and have participated in training on team building, research methods, and communication [22]. Specific demographics for the group participants are not provided due to group policy of being collaborators and not study participants. Feedback from the group was used to revise our survey for language clarity, to be culturally sensitive, and appropriate. The group also helped to revise the language in the recruitment materials.

### Data Collection

**Figure 2:**
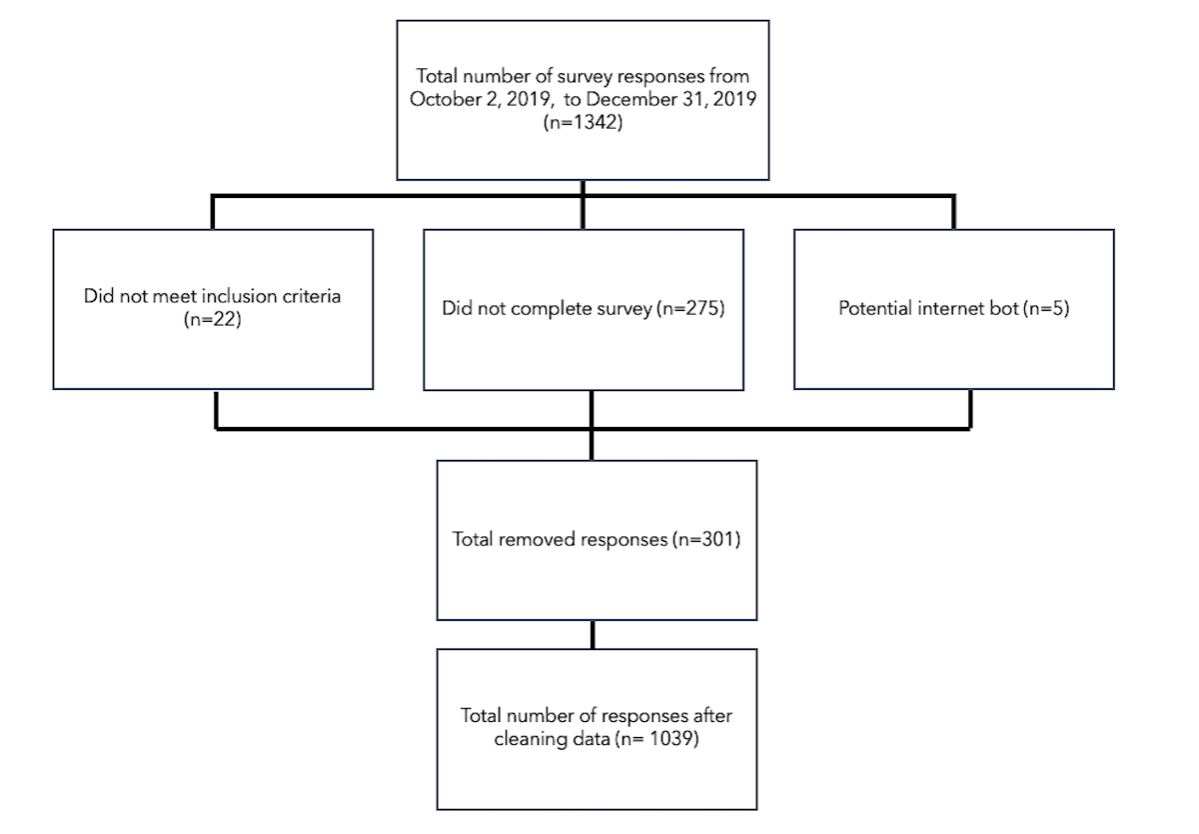
Study inclusion flow diagram including data cleaning of survey participants’ responses.

Recruitment on social media for survey participation was initially posted on October 22, 2019, and was reshared 2 times (once in each of the following months) until responses were cut off at 11:59 PM on December 31, 2019. A total of 1342 responses were counted at the end of this 2-month period. Data were screened, filtered, and cleaned before statistical analysis (Figure 2). Incomplete survey responses, those that did not meet the inclusion criteria, and those that were determined to potentially be an internet response bot (eg, random letter strings in open-ended questions) were removed. The remaining 1039 responses were used for analysis.

### Data Analysis

Quantitative data were analyzed using IBM SPSS Statistics for Windows (version 26) to create descriptive statistics including means, SDs, and frequencies.

Qualitative data from open-ended survey questions were reviewed for data transformation (eg, provide additional categories or combine responses based upon themes from open-ended “describe other” responses for check all that apply answers) and data validation (eg, explore open-ended questions for emergent themes to provide context and explanation of quantitative results) purposes [19]. Responses were reviewed to determine any commonalities that could be pooled into an existing or new category. Specifically, many of the free response options from the open-ended questions asking participants to describe the “other” response they had selected. This allowed for new response categories to be created for analysis. These original responses and revised responses are listed in Table 1. Data were further transformed as some participants’ selections were revised if open-ended answers could be synthesized into a current response option. For example, in the question investigating barriers to care, if a participant did not select “daily commitments” but did select “other” and the open-ended response was work, time, life, and so on, then the “daily commitments” was selected as a valid response and the “other” response was removed.

**Table 1:** Revised response options based upon themes in open-ended text options.

Original response options	Revised response options
**How did you hear about your OBGYN provider?**	
Friends or family Social media Covered by my insurance plan From my primary care provider Other	Internet (social media, internet search, online reviews, referral resource) In insurance network Other health care provider or practice Work or school Other
**What factors will keep you from visiting your OBGYN provider?**	
Lack of insurance coverage Cost Transportation Daily commitments Fear of diagnosis No factor would keep me from visiting Other	Lack of insurance coverage Cost Transportation Daily commitments Fear of diagnosis No factor would keep me from visiting Lack of access (distance to provider) Lack of access (availability or scheduling issues in clinic) Mistreatment by office staff Mistreatment or dismissal by provider Trauma related to visits Lack of therapeutic relationship Frustration with doctor or treatment (including disliking exam) Delaying care (wait for problems to resolve, procrastination, or worried they were overreacting) Other
**Which of the following would allow you to feel that your OBGYN provider can relate to you?**	
Same race Same geographical background Same social class Same religious background Same sex Same sexual orientation Same educational level None of these are necessary for relatability Other	Same race Same geographical background Same social class Same religious background Same gender identity Same sexual orientation Same educational level None of these are necessary for relatability Provides culturally competent care Same socio-political beliefs Same age Same life experiences Provides evidence-based care Provides compassionate care Other

In total, 2 new variables were also calculated. To determine if participants faced multiple barriers to care, a new variable was created by summing the total number of responses that were checked including the new response options. To determine if women experienced a pregnancy loss, the categorical variables responses were given a value (0=0, 1=1,…4+=5), then the number of children was subtracted from the number of times pregnant. While the exact number of pregnancy losses could not be determined, any number 1 or greater is assumed to be a pregnancy loss.

### Ethical Considerations

This project was reviewed and approved by the University of South Carolina institutional review board (Pro00092199). Informed consent was provided through an opt-in question on the first page of the survey. Through this, participants were given a description of the survey, and their willingness to participate was confirmed before continuing. No incentives were provided for participating in the study.

## Results

### Participant Demographics

The majority of respondents were highly educated (568/1039, 54.7%) had higher than a bachelor’s degree), had 1 or more children (597/1039, 57.4%), and had a mean age of 36.53 (SD 12.21) years (Table 2). Participants were given the option of selecting multiple responses for their racial or ethnic identity and a majority (901/1086, 83%) of the sample selected white. A small percentage of the sample (41/1086, 4.1%) identified as multiracial (ie, selecting more than 1 race or ethnicity). Nearly 3 in 10 (290/1086, 26.6%) of the participants experienced a pregnancy loss.

**Table 2:** Demographics of survey respondents.

Variable		Statistical values (N=1039)
Age (years), mean (SD)		36.5 (12.2)
**Gender identity, n (%)**		
	Woman	1034 (99.5)
	Nonbinary	3 (0.3)
	Other	2 (0.2)
**Race or Ethnicity^a^ (n=1086), n (%)**		
	American Indian or Alaskan native	8 (0.7)
	Asian	38 (3.5)
	Black or African American	94 (8.7)
	Hispanic or Latino/a	31 (2.9)
	Native Hawaiian or Pacific islander	2 (0.2)
	White	901 (83)
	Other	8 (0.7)
	Choose not to answer	4 (0.4)
**Marital Status, n (%)**		
	Divorced	51 (4.9)
	Married	673 (64.8)
	Separated	6 (0.6)
	Single	304 (29.3)
	Widowed	5 (0.5)
**Sexual activity, n (%)**		
	Abstinent	69 (6.6)
	Asexual	8 (0.8)
	Sex with men	907 (87.3)
	Sex with women	23 (2.2)
	Sex with both men and women	32 (3.1)
**Number of pregnancies, n (%)**		
	0	394 (37.9)
	1	188 (18.1)
	2	220 (21.2)
	3	129 (12.4)
	4+	108 (10.4)
**Number of children, n (%)**		
	No response	7 (0.7)
	0	435 (41.9)
	1	235 (22.6)
	2	271 (26.1)
	3	73 (7)
	4+	18 (1.7)
**Highest level of education**		
	No formal education	2 (0.2)
	High school diploma	52 (5)
	Vocational training	32 (3.1)
	Bachelor’s degree	385 (37.1)
	Master’s degree	243 (23.4)
	Doctoral or professional degree (JD, MD, PhD, DrPH, etc)	325 (31.3)

^a^Race or ethnicity: Race or ethnicity offers multiple response options (check all that apply).

### Relationship With Provider

Participants were asked a series of questions about their OBGYN provider. The respondents report seeing their provider for an average of 6.01 (SD 6.78) years. A total of 62% (648/1039) have seen their provider at least once per year, while 184 (17.7%) and 107 (19.9%) participants reported seeing their provider less than once per year or more than once per year, respectively. Nearly 90% either see the same provider with each visit (672/1039, 64.7%) or see an OBGYN within the same practice (253/1039, 24.4%). Approximately 15.2% (158/1039) of the reported providers are underrepresented in medicine (Black, Mexican American, American Indian, Alaska Native, and Native Hawaiian). The top 3 ways participants found their OBGYN provider were through either friends or family (495/1039, 47.6%), their insurance network (226/1039, 21.8%), or another health care provider or practice (164/1039, 15.8%). A majority of the participants (562/1039, 54.1%) stated that they will wait a few days before reaching out to their provider if they have an OBGYN-related health concern, and 14.7% (153/1039) will tough it out; yet, 1 in 4 (261/1039, 25.1%) will reach out immediately. Most participants (615/1039, 59.2%) are not afraid to share personal details with their provider; however, 27.2% (283/1039) of them do experience fear some of the time when discussing sensitive topics. A total of 863/1039 (83.0%) participants always or most of the time have a strong level of trust in their provider, and nearly all (1008/1039, 97%) reported that their provider remains professional during their appointments.

### Characteristics, Traits, and Relatability

In total, 57.4% (596/1039) of the participants indicated that it is very important or necessary that their OBGYN provider can relate to them, while only 9.2% (95/1039) of them indicated that it is of little or no importance. A total of 35% (346/1039) of the participants found none of the characteristics or traits necessary for relatability; however, the rest of the participants (675/1039, 65%) identified between 1 and 7 different traits or characteristics that could increase relatability. The most often cited characteristics (Table 3) for a provider to have that would impact relatability were same gender identity (545/675, 80.7%) followed by same race (122/675, 18.1%) and same education level (107/675, 15.9%). These results of the importance of gender identity are supported by several responses in the final open-ended questions. A word count was performed on the question inquiring about internet search terms that could be used to find the ideal OBGYN provider. The most frequent responses (n=348/3067, 7.95%) had to do with the provider’s gender (eg, woman or female OBGYN).

**Table 3:** Descriptive statistics from multiple response survey data.

Attributes		Frequency, n (%)	Percentage of cases, %
**Relatable characteristics** **(number of participants responding n=675, number of responses n=1160)**			
	Provides culturally competent care	9 (0.8)	1.3
	Other	9 (0.8)	1.3
	Provides compassionate care	32 (2.9)	5.0
	Provides evidence-based care	10 (0.9)	1.5
	Same age	15 (1.3)	2.2
	Same educational level	107 (9.2)	15.9
	Same gender	545 (47)	80.7
	Same geographical background	59 (5.1)	8.7
	Same life experiences	12 (1)	1.8
	Same race	122 (10.5)	18.1
	Same religious background	64 (5.5)	9.5
	Same sexual orientation	91 (7.8)	13.5
	Same social class	74 (6.4)	11.0
	Same sociopolitical views	9 (0.8)	1.3
**Barriers to care** **(number of participants responding n=738, number of responses n=1117)**			
	Access (availability or scheduling)	26 (2.3)	3.5
	Access (distance)	5 (0.4)	0.7
	Cost	245 (21.9)	33.2
	Daily commitments	485 (43.4)	65.7
	Delaying care	10 (0.9)	1.4
	Fear of diagnosis	128 (11.5)	17.3
	Frustration with doctor, treatment, or dislike examination	15 (1.3)	2.0
	Lack of insurance coverage	110 (9.8)	14.9
	Lack of therapeutic relationship	8 (0.7)	1.1
	Mistreatment or dismissal by provider	35 (3.1)	4.7
	Mistreated by office staff	2 (0.2)	0.3
	Other	5 (0.4)	0.7
	Transportation	38 (3.4)	5.1
	Trauma	5 (0.4)	0.7

**Figure 3:**
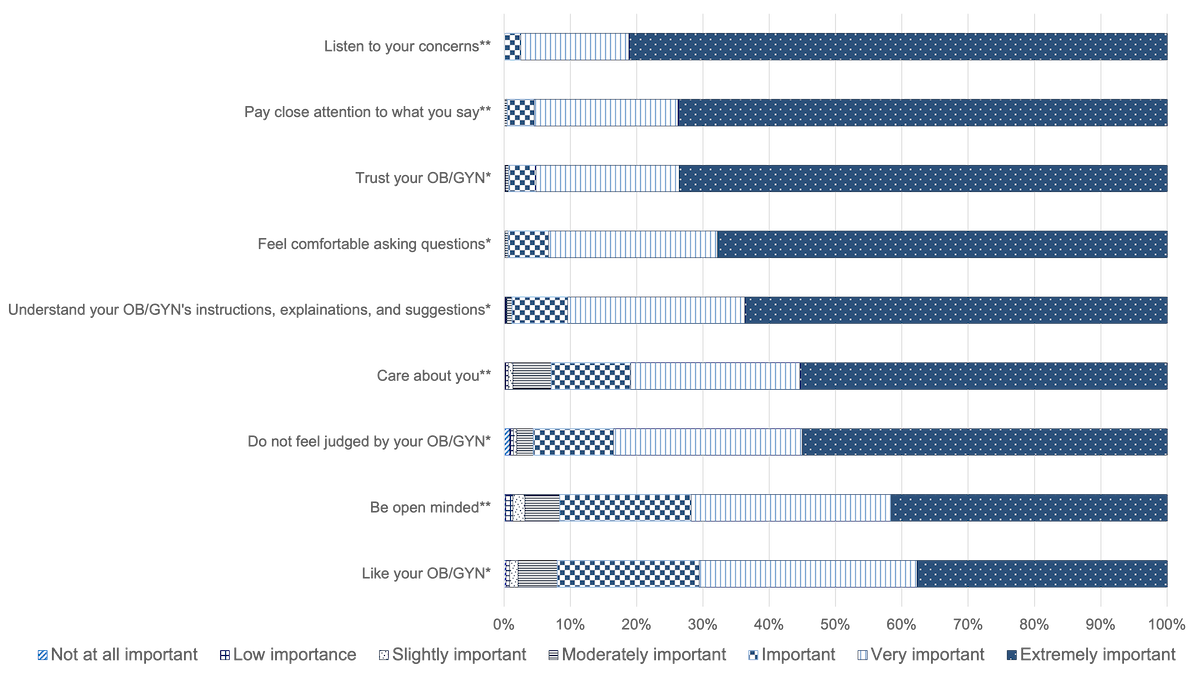
Levels of importance for factors within a therapeutic alliance between patient and provider. *: answers to question “how important is it for you to”; **: answers question “how important is it for your care provider to.”. OB/GYN: obstetrics and gynecology.

Figure 3 provides the levels of importance for each of the factors within the therapeutic alliance scale. Participants indicate that their provider listening to them is the most important part of the alliance while liking their provider is the least important factor.

### Barriers to Seeking Care

While 29.0% (301/1039) of participants report no barriers to seeking care, the rest of the participants (n=738) report between 1 and 5 total barriers to care. Table 3 indicates the percentage of respondents who cited each type of barrier. The most often cited barrier (485/735, 67.5%) to seeking care were daily commitments.

## Discussion

### Principal Findings

This study sheds new light on the preferences and values that OBGYN patients hold regarding their providers, emphasizing the critical role of relatability and trust in patient-provider relationships. Our findings underscore the paramount importance of listening skills, with patients ranking the ability to listen as more crucial than provider likeability or the delivery of medical advice. This aligns with existing literature which emphasizes empathy and listening as foundational to building trust and improving patient outcomes [1,5,23,24].

Participants in this study represent a demographic that is commonly analyzed in OBGYN research, predominantly white, cisgender women, a focus that has limited the diversity of perspectives traditionally captured in the literature [7,15,25,26]. Unlike previous studies, our findings reveal a notable preference for same-gender providers, an area where past research has shown mixed results [3,12-14,25,27]. Though evidence suggests that most patients still prioritize provider competence and communication, the role of gender concordance in specific medical specialties like obstetrics and gynecology hints at a potential shift in patient priorities within those fields [1,3,26].

Patients highly value trust, comfort, and respectful, personalized care from their providers, impacting their willingness to share personal details [1,2,5,10,23,25,28]. Our study reveals that 83% (863/1039) of participants prioritize trust, aligning with the intimacy and sensitivity inherent in OBGYN care. This focus on trust supports broader health care trends where empathy and listening skills are increasingly recognized as essential to effective patient care [6,9,23,26,28,29]. In addition, more than a quarter of our participants expressed concerns about disclosing personal information, indicating a need for providers to foster nonjudgmental and supportive environments. This aspect is particularly critical given the recent shifts toward more diverse health care teams, including the increase in female trainees and the expanding roles of nurse practitioners and primary care physicians in gynecological care, which may influence patient comfort and trust levels [3,9,12,18,30,31].

Patients highly prioritize professionalism and courtesy when selecting their OBGYN provider, aligning with the emphasis on listening skills found in this study. While studies show patients prioritize physician qualities such as experience, knowledge, and ability above all else [3], patients also consistently rank professionalism as a top factor when choosing an OBGYN provider [13,32]. Professionalism in this context encompasses traits such as courtesy, respect, and a positive bedside manner, which are closely tied to effective listening skills [1,13,32]. This study’s focus on the importance of listening skills in OBGYN care aligns with existing research highlighting the essential role of these skills in establishing trust and effective patient-clinician relationships [1,2,5,23]. By emphasizing listening as a crucial element within professionalism and courtesy, this study underscores the evolving patient expectations regarding patient-centered care, particularly in the context of increased advocacy for this approach [1,5,23].

Barriers to OBGYN care have been well documented in the scientific literature, including costs, language differences, reluctance to disclose information, inadequate insurance, transportation, discrimination, and lack of access due to geography or other structural barriers [28,33-39] While the most often discussed barriers in the literature focus on cost and access to care, our results differ indicating the greatest barrier to care is daily commitments. However, barriers of cost and insurance combined to prevent nearly half of respondents from seeking care. With a greater understanding for patient barriers, it is important to note that results of this study primarily represent the demographic of well-educated women. For this group of patients, it is expected that work or school commitments could pose difficulties in setting aside time for OBGYN appointments. By understanding a common barrier patients may face, stronger patient-physician interactions will likely be built.

### Clinical Implications

The study’s findings align with the principles of patient-centered care, suggesting avenues for educational initiatives and quality improvement efforts to enhance patient experiences and outcomes in the OBGYN setting.

The emphasis on factors such as trust, communication, and relatability aligns seamlessly with the tenants of patient-centered care and highlights that patient-provider relationships are pivotal in fostering an environment where patients feel valued and empowered in their health care journey. These findings support the scientific literature which emphasizes the significance of trust and communication in patient-provider relationships which can lead to better patient satisfaction and health outcomes [23,24,40]. The need for a high level of trust with their provider highlights the importance of strong therapeutic relationships and may be especially important for future male OBGYN providers. As a majority of participants indicated that gender concordance impacts relatability with their provider, male OBGYNs will not have the same gender advantage as their female counterparts and instead will need to focus on other desired categories such as communication and enhancing trust.

This study’s findings present opportunities for educational initiatives targeting both health care providers and patients. Providing education opportunities for OBGYN providers in effective communication skills, cultural competency, and enhancing trust could enhance their abilities to establish strong patient-provider relationships and reduce barriers to care [23,28,33,38-40]. Furthermore, providing patient education about the importance of communication, trust, and their own role in health care decision-making could encourage more active engagement in their care for patients [4-43].

Finally, the results of this study can guide quality improvement efforts within OBGYN practices through provider diversity and reducing barriers. Recognizing the importance of relatability, health care institutions can strive to diversify their provider pool to better mirror their patient populations. In addition, addressing practical barriers to care, as highlighted by the study, can be a quality improvement priority. Offering extended office hours, advanced telehealth options, and streamlining appointment processes can enhance patient access.

### Limitations and Future Directions

It was noted through a literature review that the majority of previous study participants in similar studies to this, investigating provider traits, were White heterosexual females [13,15,25,32,44]. Our study has a similar demographic majority of white females; therefore, it may be difficult to generalize patient preferences of OBGYN providers in a more diverse population. With this potential lack of generalizability to other populations, it is important to continue these studies and attempt to create a more diverse participant population. This study also suggests a strong preference for patients to have a same-sex OB-GYN provider. This may call for more research into the reasoning behind this response, as well as an investigation into patient-identified traits and suggestions to male providers.

In addition, the survey was distributed solely in English, which could exclude non–English speaking participants and limit the diversity of responses. The reliance on self-reported data introduces potential biases, including social desirability bias, where participants may respond in a manner they perceive as favorable rather than providing genuine answers. This is particularly pertinent in sensitive topics such as personal health care experiences.

Furthermore, as a cross-sectional study, the timing of the survey could influence the results. Changes in public opinion, health care policies, or societal norms that occur before or after the survey period might not be reflected in the data, affecting the study’s relevance over time. Cross-sectional designs also restrict the ability to infer causality from the associations observed, limiting the conclusions that can be drawn regarding the effects of patient preferences on health care outcomes.

Future research should incorporate expanded analyses, including detailed subgroup analyses, to explore how different demographic variables, such as age, race, and socioeconomic status, influence patient preferences and perceptions. This approach will help to address the current study’s limitations in generalizability and provide a deeper understanding of the complex factors that shape patient-provider interactions in diverse populations. In addition, amid shifting federal and state policies on pregnancy and abortion care, future research should explore how these legal changes influence patient preferences and access to OBGYN care. Investigating variations in patient attitudes across different policy environments, through longitudinal and qualitative studies, will help understand the evolving dynamics of patient-provider relationships. This research could also highlight disparities and inform interventions to enhance health care access and quality, particularly for demographics most affected by legislative changes.

### Conclusions

The relationship between an OBGYN provider and patients is one of the most intimate within medicine. Whether the interactions involve a physical examination or sensitive topic conversations, medical care in this field requires more trust and comfort than typical patient-physician relationships. The major findings of this study indicate that listening skills and building trust are valued most by patients. The data provide convincing evidence demonstrating a shift from previous research that patients can have a clear preference for OBGYN providers who share the same gender. As social climates fluctuate, women receiving reproductive health care deserve to be listened to and cared for by providers with whom they can build a strong relationship that may be influenced by pieces of one’s worn identity.

## Appendix

Multimedia Appendix 1 Survey instrument.

## Abbreviations

OBGYN: obstetrics and gynecology
REDCap: Research Electronic Data Capture

## References

[1] Srivastava A, Avan BI, Rajbangshi P, Bhattacharyya S (2015). Determinants of women's satisfaction with maternal health care: a review of literature from developing countries. BMC Pregnancy Childbirth.

[2] Dale HE, Polivka BJ, Chaudry RV, Simmonds GC (2010). What young African American women want in a health care provider. Qual Health Res.

[3] Nguyen BT, Streeter LH, Reddy RA, Douglas CR (2022). Gender bias in the medical education of obstetrician-gynaecologists in the United States: a systematic review. Aust N Z J Obstet Gynaecol.

[4] Alspach JG (2017). Because women's lives matter, we need to eliminate gender bias. Crit Care Nurse.

[5] Bogdan-Lovis E, Zhuang J, Goldbort J, Shareef S, Bresnahan M, Kelly-Blake K, Elam K (2023). Do black birthing persons prefer a black health care provider during birth? Race concordance in birth. Birth.

[6] Zhao C, Dowzicky P, Colbert L, Roberts S, Kelz RR (2019). Race, gender, and language concordance in the care of surgical patients: a systematic review. Surgery.

[7] Peterson CE, Silva A, Goben AH, Ongtengco NP, Hu EZ, Khanna D, Nussbaum ER, Jasenof IG, Kim SJ, Dykens JA (2021). Stigma and cervical cancer prevention: a scoping review of the U.S. literature. Prev Med.

[8] Miller AN, Duvuuri VNS, Vishanagra K, Damarla A, Hsiao D, Todd A, Toledo R (2024). The relationship of race/ethnicity concordance to physician-patient communication: a mixed-methods systematic review. Health Commun.

[9] Lauwers EDL, Vandecasteele R, McMahon M, De Maesschalck S, Willems S (2024). The patient perspective on diversity-sensitive care: a systematic review. Int J Equity Health.

[10] Rand LZG, Berger Z (2019). Disentangling evidence and preference in patient-clinician concordance discussions. AMA J Ethics.

[11] Mendoza-Grey S, Ramos-Muniz J, Armbrister AN, Abraído-Lanza AF (2021). Mammography screening among Latinas: does gender and ethnic patient-physician concordance matter?. J Immigr Minor Health.

[12] Mavis B, Vasilenko P, Schnuth R, Marshall J, Jeffs MC (2005). Female patients' preferences related to interpersonal communications, clinical competence, and gender when selecting a physician. Acad Med.

[13] Johnson AM, Schnatz PF, Kelsey AM, Ohannessian CM (2005). Do women prefer care from female or male obstetrician-gynecologists? A study of patient gender preference. J Am Osteopath Assoc.

[14] Zuckerman M, Navizedeh N, Feldman J, McCalla S, Minkoff H (2002). Determinants of women's choice of obstetrician/gynecologist. J Womens Health Gend Based Med.

[15] Plunkett BA, Kohli P, Milad MP (2002). The importance of physician gender in the selection of an obstetrician or a gynecologist. Am J Obstet Gynecol.

[16] Buck KS, Littleton HL (2014). Stereotyped beliefs about male and female OB-GYNS: relationship to provider choice and patient satisfaction. J Psychosom Obstet Gynaecol.

[17] Otte SV (2022). Improved patient experience and outcomes: is patient-provider concordance the key?. J Patient Exp.

[18] Smith GH, Hampton C, Brandon WP (2018). Physicians, physician extenders and health outcomes: race, gender and patient-health provider concordance in North Carolina medicaid. J Health Care Poor Underserved.

[19] Creswell JW, Plano CVL (2017). Designing and Conducting Mixed Methods Research.

[20] Davies W (2016). Insights into rare diseases from social media surveys. Orphanet J Rare Dis.

[21] Harris PA, Taylor R, Thielke R, Payne J, Gonzalez N, Conde JG (2009). Research Electronic Data Capture (REDCap)--a metadata-driven methodology and workflow process for providing translational research informatics support. J Biomed Inform.

[22] Fleming PR, Swygert MM, Hasenkamp C, Sterling J, Cartee G, Russ-Sellers R, Cozad M, Chosed RJ, Roudebush WE, Kennedy AB (2021). Patient engagement in fertility research: bench research, ethics, and social justice. Res Involv Engagem.

[23] Müller E, Zill JM, Dirmaier J, Härter M, Scholl I (2014). Assessment of trust in physician: a systematic review of measures. PLoS One.

[24] Wassenaar A, van den Boogaard M, van der Hooft T, Pickkers P, Schoonhoven L (2015). 'Providing good and comfortable care by building a bond of trust': nurses views regarding their role in patients' perception of safety in the intensive care unit. J Clin Nurs.

[25] Makam A, Mallappa Saroja CS, Edwards G (2010). Do women seeking care from obstetrician-gynaecologists prefer to see a female or a male doctor?. Arch Gynecol Obstet.

[26] Evans S, Myers EM, Vilasagar S (2019). Patient perceptions of same-day discharge after minimally invasive gynecologic and pelvic reconstructive surgery. Am J Obstet Gynecol.

[27] Janssen SM, Lagro-Janssen ALM (2012). Physician's gender, communication style, patient preferences and patient satisfaction in gynecology and obstetrics: a systematic review. Patient Educ Couns.

[28] Logan RG, Daley EM, Vamos CA, Louis-Jacques A, Marhefka SL (2021). "When Is Health Care Actually Going to Be Care?" The lived experience of family planning care among young black women. Qual Health Res.

[29] Harik L, Yamamoto K, Kimura T, Rong LQ, Vogel B, Mehran R, Bairey-Merz CN, Gaudino M (2024). Patient-physician sex concordance and outcomes in cardiovascular disease: a systematic review. Eur Heart J.

[30] Meghani SH, Brooks JM, Gipson-Jones T, Waite R, Whitfield-Harris L, Deatrick JA (2009). Patient-provider race-concordance: does it matter in improving minority patients' health outcomes?. Ethn Health.

[31] Chan KS, Bird CE, Weiss R, Duan N, Meredith LS, Sherbourne CD (2006). Does patient-provider gender concordance affect mental health care received by primary care patients with major depression?. Womens Health Issues.

[32] Piper I, Shvarts S, Lurie S (2008). Women's preferences for their gynecologist or obstetrician. Patient Educ Couns.

[33] Adler A, Biggs MA, Kaller S, Schroeder R, Ralph L (2023). Changes in the frequency and type of barriers to reproductive health care between 2017 and 2021. JAMA Netw Open.

[34] McKenney KM, Martinez NG, Yee LM (2018). Patient navigation across the spectrum of women's health care in the United States. Am J Obstet Gynecol.

[35] Mendiola M, Chu J, Haviland MJ, Meservey M, Hacker MR, Gomez-Carrion Y (2020). Barriers to care and reproductive considerations for transmasculine gender affirming surgery. Am J Obstet Gynecol.

[36] Ranji U, Salganicoff A, Rousseau D, Kaiser Family Foundation (2019). Barriers to care experienced by women in the United States. JAMA.

[37] Brown SS, Institute of Medicine (US) Committee to Study Outreach for Prenatal Care (1988). Prenatal care: reaching mothers, reaching infants. Women's Perceptions of Barriers to Care.

[38] Okoro ON, Hillman LA, Cernasev A (2020). "We get double slammed!": Healthcare experiences of perceived discrimination among low-income African-American women. Womens Health (Lond).

[39] Kingsberg SA, Schaffir J, Faught BM, Pinkerton JV, Parish SJ, Iglesia CB, Gudeman J, Krop J, Simon JA (2019). Female sexual health: barriers to optimal outcomes and a roadmap for improved patient-clinician communications. J Womens Health (Larchmt).

[40] Merenstein Z, Shuemaker JC, Phillips RL (2023). Measuring trust in primary care. Milbank Q.

[41] McCarus SD, Wiercinski K, Heidrich N (2019). Shared decision-making to improve patient engagement in minimally invasive hysterectomy. Surg Technol Int.

[42] Légaré F, Adekpedjou R, Stacey D, Turcotte S, Kryworuchko J, Graham ID, Lyddiatt A, Politi MC, Thomson R, Elwyn G, Donner-Banzhoff N (2018). Interventions for increasing the use of shared decision making by healthcare professionals. Cochrane Database Syst Rev.

[43] Stabile C, Goldfarb S, Baser RE, Goldfrank DJ, Abu-Rustum NR, Barakat RR, Dickler MN, Carter J (2017). Sexual health needs and educational intervention preferences for women with cancer. Breast Cancer Res Treat.

[44] Schnatz PF, Murphy JL, O'Sullivan DM, Sorosky JI (2007). Patient choice: comparing criteria for selecting an obstetrician-gynecologist based on image, gender, and professional attributes. Am J Obstet Gynecol.

