# Supplementary material for: Understanding the Values, Qualities, and Preferences of Patients in Their Relationships With Obstetrics and Gynecology Providers: Cross-Sectional Survey With a Mixed Methods Approach
Source: J Particip Med. 2024 Oct 16;16:e58096. doi: 10.2196/58096 (PMC11525076; doi:10.2196/58096)
Supplement: Multimedia Appendix 1 [file jopm_v16i1e58096_app1.pdf]

Patient Relationships and Communication with OB/GYN Providers

Dear Participant,

Introduction and Purpose
Our names are Anna Tarasidis, Jennifer Palomo, and Lauren Jackson. We are medical students at the University of South Carolina School of Medicine Greenville, pursuing a Doctor of Medicine (M.D.). We are working with our research mentor Dr. Ann Blair Kennedy in conducting a research study to gain knowledge of how best to serve future patients in the field of obstetrics and gynecology (OB/GYN) and would like to invite you to participate.

This study is a survey designed to gather information to better understand patient-physician relationships in OB/GYN and to discover what patients value most in their physicians. If you decide to participate, you will be asked to complete a series of questions.

In particular, you will be asked about sexual preferences, gender identity, race/ethnicity, OB/GYN experiences, and desired characteristics in physicians. You may feel uncomfortable answering some of the questions, but please note that you do not have to answer any questions you do not wish to answer. It should take approximately 8 minutes to complete all questions, and you will not be contacted again in the future unless you choose to provide your email at the end of the survey.

Confidentiality
Participation is anonymous, meaning that no one (not even the research team) will know which answers belong to you, so please do not write your name or other identifying features on any study materials.

Voluntary Participation
You are under no obligation to participate in this study, and there will be no negative consequences of ending the survey now.

Questions
We are happy to answer any questions you have about the study. You may contact us through our faculty advisor and Principal Investigator, Dr. Ann Blair Kennedy, at kenneda5@greenvillemed.sc.edu or the Institutional Review Board at 803-576-7276. Thank you for your consideration. If you would like to participate, please continue with the survey below.

With kind regards,

Anna Tarasidis, Jennifer Palomo, and Lauren Jackson
USC School of Medicine Greenville
607 Grove Road, Greenville, SC 29605
864-455-8374
kenneda5@greenvillemed.sc.edu

Are you willing to participate in this survey?
◯ Yes
◯ No

Are you currently seeing (or have you seen in the past) an OB/GYN provider for healthcare services?
◯ Yes
◯ No

Some of the survey questions are personal, but please note that all of the information will be used to improve care for patients. Are you willing to answer personal questions on potentially sensitive topics?

All results will remain completely confidential.
◯ Yes
◯ No

10%

.

We'd like to know about your relationship with your current (or past) OB/GYN

How long have you been with your OB/GYN provider (in years)? __________________________________

Do you see the same OB/GYN provider with every visit?
- ◯ Yes
- ◯ No, but I see an OB/GYN at the same practice
- ◯ No, I do not have a consistent OB/GYN or OB/GYN practice
- ◯ Other

Please define other __________________________________

How often do you see your OB/GYN provider?
- ◯ Less than once per year
- ◯ Once per year
- ◯ More than once per year

What is the race/ethnicity of your OB/GYN provider? (check all that apply)
- ☐ American Indian or Alaska Native
- ☐ Asian
- ☐ Black or African American
- ☐ Hispanic or Latino/a
- ☐ Native Hawaiian or Pacific Islander
- ☐ White
- ☐ Do not know
- ☐ Other

Please define other __________________________________

Do you have a Primary Care Provider in addition to your OB/GYN provider?
- ◯ Yes
- ◯ No

How did you hear about your OB/GYN provider?
- ◯ Friends or Family
- ◯ Social media
- ◯ Covered by my insurance plan
- ◯ From my Primary Care Provider
- ◯ Other

Please describe other __________________________________

What is the certification of your OB/GYN provider?
- ◯ MD/DO (physician)
- ◯ NP/APRN (nurse practitioner)
- ◯ I don't know
- ◯ Other

Please describe other __________________________________

20%

.

How often do you consider yourself to have a strong level of trust with your current OB/GYN provider?	◯ Never<br>◯ Some of the time<br>◯ Most of the time<br>◯ Always<br>◯ Depends on the topic
Which topics are not associated with strong levels of trust in your OB/GYN provider?	__________________________________
How often do you feel that your OB/GYN provider remains professional during your appointments?	◯ Never<br>◯ Some of the time<br>◯ Most of the time<br>◯ Always<br>◯ Depends on the topic
You answered "Depends on the topic" to the previous question, can you tell us when you have felt that your OB/GYN provider did not remain professional?	__________________________________
How often do you feel concerned about sharing personal details to your OB/GYN provider due to fear of judgment?	◯ Never<br>◯ Some of the time<br>◯ Most of the time<br>◯ Always<br>◯ Depends on the topic
What kind of topics make you fearful of judgement (ex: STDs, abortions)?	__________________________________
30%<br><br>.	
When you have an OB/GYN-related healthcare concern, which of the following best describes how you feel about reaching out to your OB/GYN provider?	◯ I will reach out to my provider immediately.<br>◯ I will give it a few days before reaching out.<br>◯ I will try to tough it out.<br>◯ I don't want to waste my provider's time.<br>◯ I don't feel that my concerns will be addressed or taken seriously.
What factors will keep you from visiting your OB/GYN provider (check all that apply)?	☐ Lack of insurance coverage<br>☐ Cost<br>☐ Transportation<br>☐ Daily commitments<br>☐ Fear of diagnosis<br>☐ No factor would keep me from visiting<br>☐ Other
Please describe other	__________________________________
How important is it that your OB/GYN provider can relate to you?	◯ Not important<br>◯ Little importance<br>◯ Important<br>◯ Very important<br>◯ Required/Necessary

Which of the following would allow you to feel that your OB/GYN provider can relate to you (check all that apply)?	☐ Same race<br>☐ Same geographical background<br>☐ Same social class<br>☐ Same religious background<br>☐ Same sex<br>☐ Same sexual orientation<br>☐ Same educational level<br>☐ None of these are necessary for relatability<br>☐ Other
Please describe other	__________________________________

40%

.

Considering your relationship with your OB/GYN provider, how important is it for your care provider to:

	Not at all important	Low importance	Slightly important	Moderately important	Important	Very important	Extremely important
Be open minded	○	○	○	○	○	○	○
Listen to your concerns	○	○	○	○	○	○	○
Pay close attention to what you say	○	○	○	○	○	○	○
Care about you	○	○	○	○	○	○	○

50%

.

Considering your relationship with your OB/GYN provider, how important is it for you to:

	Not at all important	Low importance	Slightly important	Moderately important	Important	Very important	Extremely important
Like your OB/GYN	◯	◯	◯	◯	◯	◯	◯
Trust your OB/GYN	◯	◯	◯	◯	◯	◯	◯
Do not feel judged by your OB/GYN	◯	◯	◯	◯	◯	◯	◯
Feel comfortable asking questions of your OB/GYN	◯	◯	◯	◯	◯	◯	◯
Understand your OB/GYN's instructions, explanations, and suggestions	◯	◯	◯	◯	◯	◯	◯

60%

.

We would like to know a little about you:

How many times have you been pregnant?

- ◯ 0
- ◯ 1
- ◯ 2
- ◯ 3
- ◯ 4
- ◯ 4+

How many children do you have?

- ◯ 0
- ◯ 1
- ◯ 2
- ◯ 3
- ◯ 4
- ◯ 4+

70%

.

Your Age: __________________________________

What is your marital status?

- ◯ Single
- ◯ Married
- ◯ Separated
- ◯ Divorced
- ◯ Widowed

What sex were you assigned at birth (ie, on your birth certificate)?

- ◯ Female
- ◯ Male

Which best describes your gender identity?

- ◯ Female
- ◯ Male
- ◯ Non-Binary
- ◯ Transgender (F to M)
- ◯ Transgender (M to F)
- ◯ Prefer not to say
- ◯ Other

Please define other __________________________________

Which best describes your sexual activity?

- ◯ Abstinent
- ◯ Asexual
- ◯ Sex with men
- ◯ Sex with women
- ◯ Sex with both men and women

What is your race/ethnicity (check all that apply):

- ☐ American Indian or Alaska Native
- ☐ Asian
- ☐ Black or African American
- ☐ Hispanic or Latino/a
- ☐ Native Hawaiian or Pacific Islander
- ☐ White
- ☐ Other
- ☐ Choose not to answer

Please define other __________________________________

What is your highest level of education?	◯ No formal education<br>◯ High school diploma<br>◯ Vocational training<br>◯ Bachelor's degree<br>◯ Master's degree<br>◯ Professional degree (JD, MD, etc.)<br>◯ Doctoral degree (PhD, PsyD, DrPH, etc.)
80%<br><br>.	
What words would you type into Google to find the ideal OB/GYN provider (ex: woman, in network, empathetic)?	__________________________________
90%<br><br>.	
Is there anything else we should know about you or your relationship with your OB/GYN provider?	__________________________________
If you are interested in the results of this study please provide your email address	____________________________
100%<br><br>.	
